# Responding to COVID-19 in eye health

**Published:** 2020-09-01

**Authors:** Victor Hu, N Venkatesh Prajna, Simon Arunga, Elmien Wolvaardt, Fatima Kyari, Astrid Leck, Esmael Habtamu, Heiko Philippin

**The COVID-19 pandemic has brought enormous challenges, and its repercussions have been felt around the globe, including in eye care.**

**Figure:**
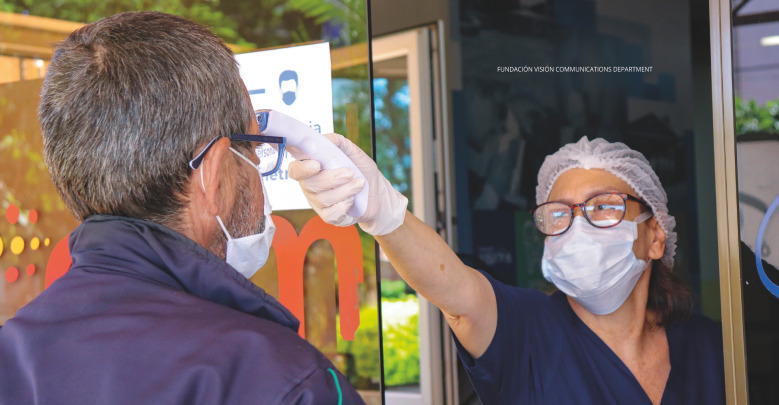
Patients have to answer questions about COVID-19 symptoms and submit to a temperature check before entering the hospital. **PARAGUAY**

Millions of people have been infected with the SARS-CoV-2 virus and many have become seriously ill, threatening to overwhelm the ability of health systems to cope. Many lives have been lost – including those of health care workers.

Countries have adopted different strategies to reduce transmission, including:

- Encouraging hand washing, the wearing of masks in public places, and social distancing, such as staying 1–2 metres away from others in public
- Containment of areas where there are outbreaks by restricting travel into and out of affected countries, for example
- Enforcing quarantine or lock-down measures.

## The human cost

The virus itself, and the measures taken to contain its spread, have had a profound impact on individuals and the community, including long-term social isolation and massive disruption to businesses and the economy. The livelihoods of many have been put at risk and others have been forced into poverty.

Existing inequalities in gender, ethnicity, working conditions, and socioeconomic status have been exposed and exacerbated by the pandemic across the globe. For example, people living in overcrowded accommodation or informal urban settlements are at higher risk of contracting and spreading the virus. Daily wage earners are often forced to continue working even if they develop symptoms of an infection; if not, they risk falling into poverty. Women, who make up 70% of the global health workforce worldwide,^1^ are likely to bear a disproportionate proportion of domestic labour, including looking after children when schools are closed.

Health care workers are facing many personal and professional challenges while working on the front line of the efforts against this pandemic.

It should come as no surprise that mental health and wellbeing have emerged as an urgent priority; not just for health care workers, but also for patients and the wider community.

People with disabilities also face challenges, whether this is with the practicalities of hand washing or when communicating with health workers who are wearing face masks. It is therefore vital that we include people with disabilities when planning and communicating about the changes to eye services during the pandemic.

## Adapting eye services

Eye health providers, along with other sectors, have had to reshape how services are provided while responding to a constantly evolving situation, full of uncertainties; this includes reducing services and deciding which patients to see, and when.

The virus has officially reached almost every country, and there has been significant variation in national measures and how long these will remain in place. We present various case studies in this issue to show how different eye units around the world are responding to the challenge and dealing with the reopening of services. More case studies and articles are available via the *Community Eye Health Journal* app (**bit.ly/CEHJ-app**) and on our website (**cehjournal.org**).

Vital tools to protect health workers and patients from COVID-19 include cleaning and disinfection, the correct use of personal protective equipment, and handwashing using soap and water and/or hand sanitiser. At times, however, global supply chains have been unable to meet the demand for PPE, which has resulted in shortages in many countries across the globe. This issue features a collection of hands-on tips for making the most of personal protective equipment, cleaning and disinfecting the hospital environment, and producing face shields and alcohol-based hand sanitiser.

**Guidelines and useful websites**

Guidelines are being updated continually as circumstances change and more becomes known about COVID-19. We encourage you to visit these websites, and those mentioned in the articles in this issue, to stay up to date.

- International Agency for the Prevention of Blindness (IAPB). COVID-19 and Eye Health. **bit.ly/IAPBcov19**
- World Health Organization (WHO). Country & Technical Guidance – Coronavirus disease (COVID-19). **bit.ly/WHOcov19**
- International Council for Ophthalmology (ICO). Coronavirus Information for Ophthalmologists. **bit.ly/ICOcov19**
- Royal College of Ophthalmologists. COVID-19 Clinical Guidelines. **bit.ly/RCOpth**
- American Academy of Ophthalmology. Coronavirus and Eye Care. **bit.ly/AAOcov19**
- World Council of Optometry. COVID-19 news. **bit.ly/WCOcov19**

We must take whatever measures are needed to protect everyone in the eye care team from COVID-19, including non-clinical or non-medical workers such as security guards, porters, and cleaners, whose work is vital for safe eye care delivery and yet often invisible.

SARS-CoV-2 is a new virus, and we are continuously learning about it. Guidelines are therefore changing constantly to reflect new research as it comes in. We encourage you to read and follow the national guidelines in whichever country you are based, and to visit the websites in the panel to stay up to date with new developments.

**“SARS-CoV-2 is a new virus, and we are continuously learning about it. Guidelines are therefore changing constantly to reflect new research as it comes in.”**

In this time, we need to carefully appraise the information available and make measured, rational judgements. We still have the responsibility and privilege of providing the best care we can to our patients, no matter what the situation is. We also need to continue to integrate our services into universal health care; more so now than ever.

The COVID-19 pandemic has pushed the world to its limits in many respects. But, along with the challenges, it has also brought opportunities to rethink and re-evaluate practices and fast-forward innovations, including the use of videoconferencing tools for triage and teaching. The inspiration for this special CEHJ issue came from the recent ICEH online conference “Ophthalmology and COVID-19 in African Units” (**bit.ly/COV19videoconf**) attended by more than 270 eye care professionals, predominantly from sub-Saharan Africa. This online event is one among several other examples that showed we can stay connected and search for solutions across the globe, together. We hope that this issue will provide you with the information and tools you need to keep yourself and your patients safe during this time, and to serve communities in need of accessible, high-quality eye care.
